# Male partners’ involvement in pregnancy related care among married men in Ibadan, Nigeria

**DOI:** 10.1186/s12978-020-0850-2

**Published:** 2020-01-28

**Authors:** Olayinka Falade-Fatila, Ayodeji Matthew Adebayo

**Affiliations:** 1 Department of Nursing, University College Hospital, Ibadan, Nigeria; 2 Department of Community Medicine, College of Medicine, University of Ibadan, Ibadan, Nigeria

**Keywords:** Male involvement, Pregnancy related care, Antenatal care, Postnatal care, Newborn care

## Abstract

**Background:**

Maternal death remains a public health burden in the developing countries including Nigeria and the major causes are pregnancy related. Lack of male involvement in pregnancy related care is one of the contributing factors. Previous studies on male involvement focused on family planning services and were majorly targeted at women. This study, therefore, was carried out to assess the knowledge, perception and involvement of male partners in pregnancy related care among married men in Ibadan, Nigeria.

**Methods:**

A cross sectional study was conducted using a four-stage sampling technique to select 367 married men in an urban community in Ibadan. A semi-structured, interviewer-administered questionnaire was used to obtain information on the knowledge, perception and involvement of respondents regarding pregnancy related care. Responses to questions on knowledge of pregnancy related care were converted to a 33-point scale. Scores greater than or equal to the mean knowledge score (26.2) were categorized as good knowledge of pregnancy related care. Similarly, responses to involvement in pregnancy related care questions were converted to a 24-point scale with scores greater than or equal to the mean (15.1) classified as good involvement in pregnancy related care. Data were analyzed using descriptive statistics and association between qualitative variables was established using Chi-square test at *p* < 0.05.

**Results:**

Sixty-three percent had good knowledge of pregnancy related care. Majority believed that they had roles to play in their partners’ care during pregnancy (89.9%), labor and delivery (92.9%), and in newborn care (97.5%). Overall, 56.9% had good involvement in pregnancy related care. About 20% followed their partners to antenatal care (19.6%) and postnatal (19.9%) clinics. A significantly higher proportion of respondents with good knowledge accompanied their partners for antenatal care (*p* = 0.008) and postnatal care clinic (*p* = 0.014); participated in birth preparedness (*p* < 0.001) and assisted with newborn care (*p* < 0.001). Job demands, social stigma and long waiting time at the health facilities were reasons highlighted for non-involvement in pregnancy related care.

**Conclusions:**

The study revealed gaps in knowledge and involvement in pregnancy related care. There is a need for reproductive health policy review to strongly emphasize the need for involvement of male partners in reproductive health issues including pregnancy related care.

## Plain English summary

Deaths among mothers remain a serious burden in the developing countries, including Nigeria. Lack of male involvement in pregnancy related care is one of the contributing factors. This study was carried out to assess the knowledge, perception and involvement of male partners in pregnancy related care among married men in Ibadan, Nigeria.

Three hundred and sixty-seven married men were selected to participate in the study from an urban community in Ibadan. A questionnaire was used to obtain information on the knowledge, perception and involvement of respondents regarding pregnancy related care like antenatal and postnatal care, labour, delivery and newborn care. Data were analyzed and presented in frequency and proportions and association between variables of interest were tested using appropriate statistical measures.

Sixty-three percent had good knowledge of pregnancy related care. Majority believed that they had roles to play in their partners’ care during pregnancy (89.9%), labor and delivery (92.9%), and in newborn care (97.5%). Overall, 56.9% had good involvement in pregnancy related care. About 20% each followed their partners to antenatal care (19.6%) and postnatal (19.9%) clinics. A significantly higher proportion of respondents with good knowledge were involved in accompanying their partners for antenatal care and postnatal care; participated in birth preparedness and assisted with newborn care. Job demands, social stigma and long waiting time at the health facilities were reasons highlighted for non-involvement in pregnancy related care.

The study revealed gaps in knowledge and men’s involvement in pregnancy related care.

## Background

Maternal death remains a public health burden in the developing countries, including Nigeria [26]. A maternal mortality ratio of 239/100,000 live births in developing countries as compared to 12/100,000 live births in developed countries is still unacceptably high [38]. A high burden of maternal deaths (99%) is borne by developing countries [38] with more than half occurring in sub-Saharan Africa [39]. Over one third of all maternal deaths worldwide were in Nigeria and India in 2015, with an approximate 58,000 maternal deaths (19%) and 45,000 maternal deaths (15%), respectively [39]. The maternal mortality ratio of Nigeria is 576 per 100,000 live births [26]. Although the health of mothers is determined by many factors including socio-economic status and environmental factors, one important factor that has been neglected over the years is the role of men [24].

The International Conference on Population and Development (ICPD), held in Cairo, Egypt in 1994 and Fourth World Conference on Women, held in Beijing, China in 1995 recognized the importance of the role of men in promoting their own and their partners’ sexual and reproductive health. The ICPD urged that special efforts be made to emphasize men’s shared responsibility and to promote their active involvement in maternal care [36]. In spite of this, pregnancy and childbirth continue to be regarded as exclusively women’s affairs in most African countries [11, 12]. Men generally do not accompany their partners to antenatal and postnatal care or family planning services and are not expected to attend the labor or birth of their children [11, 24]. The lack of participation of men in antenatal, postnatal, newborn or post-abortion care may be because they do not benefit from any information given by health providers regarding the health of the mother and the baby, or about their roles in it.

The belief that men should be involved in maternal care of their pregnant partners has gained momentum and has become important because of the realization that men’s behavior can significantly affect the health outcomes of the women and babies [11]. Various studies have emphasized how men’s role can contribute to better outcomes of their pregnant wives [18]. Morhason-Bello et al. [20] reported that 86% of antenatal clients in University College Hospital, Ibadan, preferred their husbands as companions during labor. Another study conducted by the United Nations Population Fund (UNFPA) in Kenya found that husbands greatly influence women’s decisions to use reproductive health services such as family planning [37]. These emphasize the fact that men have a critical role to play in safeguarding the health of women during pregnancy, labor and beyond [27] and there is a tendency to overlook this relevance.

Gender equality is another reason for men involvement in pregnancy related care (PRC). In most families in Katmandu, Nepal, the men are empowered financially and are the main decision-makers in all issues including pregnancy care [4]. This experience is similar in countries like Nigeria where patriarchal family setting is the norm. They may channel this opportunity to ensure that their pregnant wives seek maternity services or arrange for skilled care during delivery. Gender inequality is a fundamental cause of women’s constrained access to health services. Men can support their partners to navigate gendered barriers to care-seeking and optimal home care practices. Men’s behavior influences the reproductive health of both men and women and the health of their children [2]. Yet men are often unable to make informed choices because they have not been included in reproductive, maternal and child health services and education [13]. For men to support their wives to make the right decision regarding utilization of sexual and reproductive health (SRH) services, they need to understand the importance of and their roles in pregnancy related care.

Previous studies on male involvement focused on family planning services and antenatal care, especially following partners to antenatal care clinics and these studies were majorly targeted at women. Understanding men’s knowledge and feelings about their involvement in their partners’ obstetric care can inform interventions to reduce the burden of maternal morbidity and mortality in Nigeria. This study was conducted to assess the perception, attitude and involvement of men in pregnancy related health care. This will help in understanding men’s disposition and serve as a guide in designing reproductive health targeted programs.

## Materials and methods

This descriptive cross-sectional study was conducted amongst married men. The study location was the Idikan community in Ibadan, the capital of Oyo State, located in the southwestern part of Nigeria and is dominated by the Yoruba speaking ethnic group. The study population included married males from the age of 18 years upwards residing within the Idikan community. A minimum sample size (n) of 335 was estimated using the Leslie Kish formula (Zα^2^pq/d^2^) for single proportion in descriptive studies. The parameters were the standard normal deviate corresponding to the probability α (Z_α_) of 1.96, the proportion (p) of married men who participated in antenatal, delivery and post-natal care (32.1%) from a previous study [11] and degree of accuracy (d) of 0.05. The minimum sample estimate was increased to 373 with allowance for 10% non-response. A multi-stage (four-stage) sampling technique was used to select respondents:

### Stage 1

One of the five Local Government Areas (LGA) in Ibadan metropolis was selected by balloting.

### Stage II

The number of wards in the selected LGA was ascertained from the local government secretariat. One ward was selected randomly by balloting.

### Stage III

One settlement was selected from the selected ward by balloting.

### Stage IV

All the houses in the settlement were identified using the primary healthcare centre (PHC) numbers. In houses where there were more than one household, one household was randomly selected by balloting and a married man (18 years and above) who met the inclusion criteria was interviewed. In households where there was more than one married man, a random selection by balloting was employed to select one married man.

A semi-structured, interviewer-administered questionnaire was used to obtain information among 375 respondents. Section A of the questionnaire covered socio-demographic information (age, educational level, occupation, religion, tribe etc); Section B covered respondents’ knowledge on PRC (awareness and meaning of various maternal health care services such as antenatal care (ANC), postnatal care (PNC), newborn care and post-abortion care); Section C included perception of their roles as regards to PRC (agree, indifferent or disagree on issues related to men supporting their wife in pregnancy related services); Section D covered involvement of respondents in PRC services (previous involvement in pregnancy related services such as ANC, PNC, newborn care and post-abortion care). Questionnaires were administered by two trained research assistants.

The questions on knowledge and involvement of the respondents regarding PRC were scored and knowledge and involvement scores were computed for individual respondent. Mean score was taken as cut-off values to categorize into good or poor knowledge (of) and men’s involvement in pregnancy related care [15, 29]. Regarding knowledge, each incorrect response was scored 0 while correct was scored 1. Minimum and maximum obtainable scores on knowledge of PRC were 0 and 33 points. Scores greater equal to or greater than the mean knowledge score (26.2) was categorized as good knowledge of PRC. Involvement in the pregnancy related items was scored 1 and lack of involvement 0. Similarly, responses to involvement in PRC questions gave a cumulative mimimum and maximum obtainable scores of 0 and 24 points. Scores greater than or equal to the mean (15.1) classified as good involvement in PCR.

The data were analyzed using Statistical Package for Social Sciences (SPSS) version 21. Chi-square was used to test for statistical significance between categorical variables and the level of significance was set at 0.05. Ethical clearance was obtained from the Ethical Committee, Oyo State Ministry of Health Research Ethical Review Committee and verbal informed consent was sought from each respondent. Only those who consented were included in the study.

## Results

**Fig. 1:**
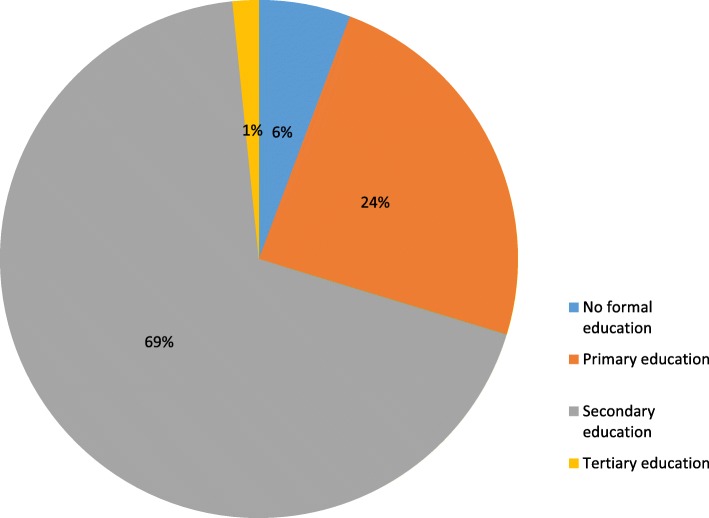
Distribution of respondents by educational status

A total of 375 households were visited from which one married man per household was interviewed and 367 respondents completed the study giving a response rate of 97.9%. The mean age of respondents was 37.5 ± 7.5 years with the highest proportion (54.5%) within the ages of 25 and 39 years. Most (62.9%) of the respondents were Muslims, 62.7% were artisans and 88.8% were of Yoruba tribe. Majority (92.6%) earned a monthly income of N50, 000 ($164) or less and 88.0% of them reported a monogamous relationship. Three hundred and fifteen (85.8%) had between one and four children (Table 1). Most (68.7%) of the respondents had secondary education and about one quarter (89) had a primary education (Fig. 1).

**Table 1:** Socio–demographic characteristics of respondents

Variables (*N* = 367)	*n*	Percentage
Age group (years)		
< 24	7	1.9
25–39	200	54.5
≥ 40	151	41.1
No response	9	2.5
Mean age	37.5 ± 7.5	
Religion		
Christianity	134	36.5
Islam	231	62.9
Traditional	2	0.6
Ethnicity		
Yoruba	326	88.8
Igbo	28	7.6
Hausa	9	2.5
Others	4	1.1
Occupation		
Artisan	230	62.7
Trader	125	34.0
Others	11	3.3
Average income (N)		
≤ 50,000	340	92.6
> 50,000	27	7.4
Family type		
Monogamous	323	88.0
Polygamous	44	12.0
Educational status of the mother of respondents’ last child		
No formal education	20	5.4
Primary education	89	24.3
Secondary education	254	69.2
Tertiary education	4	1.1
Occupation of the mother of respondents’ last child		
Artisan	101	27.5
Trader	235	64.1
Housewife	13	3.5
Others	18	4.9
Number of children		
≤ 4	258	70.3
> 4	91	24.8
No response	18	4.9

Respondents who had ever heard of ANC accounted for 94.8% and more than half (55.9%) correctly reported that the first ANC visit should take place in the first trimester of pregnancy. Respondents reported the following as reasons why pregnant women attend antenatal care: medical check-up (34.6%), to receive advice on pregnancy care (20.7%), determination of baby’s sex (12.5%) and to avoid pregnancy complications. Majority of the respondents knew the dangers that can occur during and after delivery such as high blood pressure (98.9%), chills and high fever (97.8%), swollen legs or face (75.7%) and baby could stop crying (99.7%). A large proportion of the respondents knew HIV and Packed Cell Volume as part of the regular screening tests routinely done during pregnancy, (79.6%) and (73.6%) respectively. Slightly above half (189) of the respondents did not know Venereal Disease Research Laboratory test as one of the regular screening tests required during pregnancy. Two hundred and thirty-two (63.2%) of the respondents said sex should not recommence 6 weeks after delivery and 25.6% reported new born care is solely the responsibility of the woman. There were 88.8% of the respondents who agreed with the fact that unsafe abortion can be complicated by infertility (subsequent inability to conceive), rest and exercise are essential during pregnancy (98.4%) and after delivery (72.5%). Overall, majority (62.9%) had good knowledge of PRC while 136 (37.1%) had poor knowledge (Table 2).

**Table 2:** Distribution of respondents regarding knowledge of pregnancy related issues and overall knowledge rating

Distribution of respondents regarding knowledge of pregnancy related issues		
Variables (*N* = 367)	Yes n (%)	No n (%)
Routine screening tests requested during pregnancy		
HIV	292 (79.6)	75 (20.4)
Venereal Disease Research Laboratory Test (Syphilis)	178 (48.5)	189 (51.5)
Packed Cell Volume	270 (73.6)	97 (26.4)
Other pregnancy related issues		
Tetanus vaccine is not given in pregnancy	120 (32.7)	247 (67.3)
One of the purposes of family planning is to space birth	349 (95.1)	18 (4.9)
Unsafe abortion can be complicated by infertility	326 (88.8)	41 (11.2)
Rest and exercise are essential during pregnancy	361 (98.4)	6 (1.6)
Rest and exercise are essential after delivery	266 (72.5)	101 (27.5)
Newborn care is solely the responsibility of the woman	94 (25.6)	273 (74.4)
Sex should recommence 6 weeks after delivery	135 (36.8)	232 (63.2)
Good nutrition is needed for maintaining a healthy status of the mother and the child.	362 (98.6)	5 (1.4)
After an abortion, sexual relation should be avoided until bleeding stops.	350 (95.4)	17 (4.6)
Knowledge rating on pregnancy related care		
Variable (*N* = 367)	Frequency	Percentage
Knowledge of pregnancy related care		
Good	231	62.9
Poor	136	37.1

Almost all (98.7%) the respondents said men do have important roles to play during and after pregnancy. Only 92 respondents (25.1%) agreed that men should attend ANC clinics with their wives. More than two-third (69.8%) of the respondents disagreed that following their wives to the clinic is a form of idleness. More than half (58.3%) of respondents said men can assist with changing diaper for their infant while just 151 (41.1%) said men’s involvement in infant stimulation by singing is a form of idleness. Majority (91.5%) of respondents disagreed with post abortion care being primarily a woman’s concern (Table 3).

**Table 3:** Distribution of respondents’ perception of certain roles men can be involved in pregnancy related care

Variables (*N* = 367)	Agree *n* (%)	Undecided *n* (%)	Disagree *n* (%)
Men do not have an important role to play during pregnancy since it is a woman’s issue to carry the pregnancy	3 (0.8)	2 (0.5)	362 (98.7)
Men have roles to play in the care of a woman during and after pregnancy	362 (98.6)	0 (0.0)	5 (1.4)
There is need for men to be educated on what roles they can play to ensure the woman is healthy before and during pregnancy	325 (88.6)	4 (1.1)	38 (10.3)
Men should be involved in the care of the new born	361 (98.4)	0 (0.0)	6(1.6)
Providing money to take care of expenses that arise from pregnancy related issues is the only role of men in pregnancy related care	79 (21.5)	1 (0.3)	287 (78.2)
The man needs to join the wife in taking decisions that concerns pregnancy	357 (97.3)	1 (0.3)	9 (2.4)
Men should attend antenatal clinics with their wives	92 (25.0)	19 (5.2)	256 (69.8)
Following your wife to the clinic is a form of idleness	122 (33.3)	13 (3.5)	232 (63.2)
Men can assist with diaper changing for the infant	214 (58.3)	8 (2.2)	145 (39.5)
Men who are involved in infant stimulation like singing are idle	151 (41.1)	6 (1.6)	210 (57.3)
Post-abortion care is primarily a woman’s concern	27 (7.3)	4(1.1)	336 (91.6)
Men do not have an important role to play during pregnancy since it is a woman’s issue to carry the pregnancy	3 (0.8)	2 (0.5)	362 (98.7)

Respondents were asked about perception of their roles in the care of their partners during pregnancy and labour; after delivery and in newborn and post-abortion care. Majority of the respondents believed they have roles to play during pregnancy (89.9%), during labour, after delivery (92.9%) and in newborn care (97.5%). Half (55.1%) of the respondents did not think they have a role to play in abortion and post-abortion care. Amongst the roles reported were domestic support, financial support and spiritual support (Table 4).

**Table 4:** Perception of respondents regarding specific roles in pregnancy related care

Variables	Frequency	Percentage
Perception of your role in the care of your partner during pregnancy (*N* = 367)		
Yes	330	89.9
No	37	10.1
Roles perceived to be needful during pregnancy (*N* = 330)		
Domestic support only	183	49.9
Financial support only	98	26.7
Both	49	13.4
Reasons for non-support during pregnancy (*N* = 37)		
Not always around	10	2.7
Getting support from family members and older children	13	3.5
Assistance from housemaid	1	0.3
No response	13	3.5
Perception of your role in the care of your partner during labour and after delivery (*N* = 367)		
Yes	340	92.6
No	18	4.9
No response	9	2.5
Roles perceived to be needful during labour and after delivery (*N* = 340)		
Spiritual support only	169	49.7
Financial support only	58	17.1
Take her to the hospital and spiritual support	30	8.8
Financial and spiritual support	83	24.4
Reasons for non-support during labour and after delivery (*N* = 18)		
Don’t know what can be done	12	66.7
No response	6	33.3
Perception of your role in the care of the newborn (*N* = 367)		
Yes	358	97.5
No	9	2.5
Roles perceived to be needful in newborn care (*N* = 358)		
Domestic support only	115	32.1
Financial support only	216	60.3
Both	20	5.6
No response	7	2.0
Reasons for non-support in the care of the newborn (*N* = 9)		
Not always around	1	11.1
No response	8	88.9
Perception of your role in abortion and post-abortion care (*N* = 367)		
Yes	165	45.0
No	202	55.0
Roles perceived to be needful in post-abortion care (*N* = 165)		
Take her to the hospital	35	21.2
Adequate rest	18	10.9
Assist to get her medication	27	16.4
All	79	47.9
No response	6	3.6
Reasons for non-support in post-abortion care (*N* = 202)		
Abortion is a sin	38	18.8
Will never experience it because we practice family planning	4	2.0
No response	160	79.2

A large proportion (87.7%) of the respondents were involved more in the support of their partners in post-natal care while above half were involved in labour and delivery care (59.4%), ANC (54.5%) and newborn care (51.0%). However, more than half (56.9%) of the respondents had good involvement in pregnancy related care (Table 5).

**Table 5:** Distribution of respondents’ involvement in the domains of pregnancy related care

Variables	Involvement in pregnancy related care	
	Good n (%)	Poor n (%)
Antenatal care	200 (54.5)	167 (45.5)
Postnatal care	322 (87.7)	45 (12.3)
Newborn care	187 (51.0)	180 (49.0)
Labour and delivery	218 (59.4)	149 (40.6)
Birth preparedness	294 (80.1)	73 (19.9)
Overall	209 (56.9)	158 (43.1)

Seventy-two (19.6%) and 19.9% of the respondents accompanied their wives to antenatal and postnatal clinic visits respectively while 83 (23.4%) reported that they assisted their wives in the kitchen to prepare food when she was pregnant. Overall, more than half (56.9%) of the respondents had good involvement in pregnancy related care. Majority (87.7%) of the respondents were involved more in the support of their partners in post-natal care while just above half were involved in labour and delivery care (59.4%) and ANC (54.5%) (Table 6).

**Table 6:** Distribution of respondents on their involvement in pregnancy related care

Variable (*N* = 367)	Yes *n* (%)	No *n* (%)
Attended counseling sessions with your wife before achieving pregnancy	65 (17.7)	302 (82.3)
Gave her permission to attend the ANC clinic	351 (95.6)	16 (4.4)
Accompanied her for ANC	72 (19.6)	295 (80.4)
Followed her to postnatal clinic visits	73 (19.9)	294 (80.1)
Accompanied partner to labor ward	218 (59.4)	149 (40.6)
Provided money for investigations such as ultrasound scan	360 (98.1)	7 (1.9)
Made money available for expenses on pregnancy related services	349 (95.1)	18 (4.9)
Provided good and adequate food	361 (98.4)	6 (1.6)
Reminded her to take her routine ANC drugs	331 (90.2)	36 (9.8)
Reminded her to go for her clinic visits when she is pregnant	318 (86.6)	49 (13.4)
Took care of domestic chores when she was pregnant	250 (68.1)	117 (31.9)
Gave emotional support during times of discomfort and tiredness	356 (97.0)	11(3.0)
Assisted in the kitchen to prepare food	86 (23.4)	281(76.6)
Helped to buy things in the market	234 (63.8)	133 (36.2)
Arranged means of communication in case of emergencies (e.g. buy her a phone)	292 (79.6)	75 (20.4)
Arranged for transport to take her to the clinic	228 (62.1)	139 (37.9)
Saved money towards her delivery in case of emergency and referral	336 (91.6)	31 (8.4)
Reminded her to take her routine ANC drugs	331 (90.2)	36 (9.8)
Reminded her to go for her clinic visits when she is pregnant	318 (86.6)	49 (13.4)
Stayed home with children	158 (43.1)	209 (56.9)
Bought baby’s clothes	339 (92.4)	28 (7.6)
Got some other person to take care of the home and the children during the mother’s absence	175 (47.7)	192 (52.3)
Assisted with changing of diaper	159 (43.3)	208 (56.7)
Helped to ensure that mother is comfortable while breastfeeding the Infant	116 (31.6)	251 (68.4)
Discussed with partner before pregnancy is achieved	132 (36.0)	235 (64.0)
Decided with wife whether or not to attend ANC	252 (68.7)	115 (31.3)

A significantly higher proportion of respondents with good knowledge of PRC were involved in accompanying their partners for ANC visits (*p* = 0.008), arranging means of communication for their partners in case of emergencies (*p* < 0.001), following their partners to the PNC clinic for her visit (*p* = 0.014), accompanying her to labour ward (*p* = 0.050) and assisting with changing of diaper (*p* < 0.001) compared with their counterparts with poor knowledge. Regarding taking care of domestic chores when the partner is pregnant and attending counseling session with the partner before achieving pregnancy, there were no statistically significant associations with the knowledge of PRC (Table 7).

**Table 7:** Association between knowledge of pregnancy related care among respondents and their involvement in pregnancy related care

Variables	Knowledge of pregnancy related care		*X*^2^	*p*-value
	Good n (%)	Poor n (%)		
Attended counseling sessions with your wife before achieving pregnancy				
Yes	42 (18.2)	23 (16.9)	0.095	0.758
No	189 (81.8)	113 (83.1)		
Accompanied for ANC				
Yes	55 (23.8)	17 (12.5)	6.943	0.008*
No	176 (76.2)	119 (87.5)		
Took care of domestic chores when she was pregnant				
Yes	153 (66.2)	97 (71.3)	1.021	0.312
No	78 (33.8)	39 (28.7)		
Arranged means of communication in case of emergencies (e.g. buy her a phone)				
Yes	202 (87.4)	90 (66.2)	23.817	< 0.001*
No	29 (12.6)	46 (33.8)		
Followed her to PNC clinic for her visit				
Yes	55 (23.8)	18 (13.2)	6.007	0.014*
No	176 (76.2)	118 (86.8)		
Accompanied partner to labour ward				
Yes	146 (63.2)	72 (52.9)	3.738	0.050*
No	85 (36.8)	64 (47.1)		
Assisted with changing of diaper				
Yes	125 (54.1)	34 (25.0)	29.547	< 0.001*
No	106 (45.9)	102 (75.0)		

*Significant at 5%

Concurrent job demand (68.7%), social stigma (51.8%) and long waiting time at the health facilities (50.7%) were the reasons reported by respondents as being responsible for lack of involvement of men in pregnancy related care.

## Discussion

This study was carried out to assess the knowledge, perception and involvement of married men in PRC in in Ibadan, Nigeria.

This study found that most (62.9%) of the respondents had good knowledge of pregnancy related care. This is similar to the finding of Nasreen et al. [28], in a comparative study carried out in rural Bangladesh to assess men’s awareness and knowledge of maternal, neonatal and child health among men. In the Bangladesh study, a relatively higher proportion of the respondents were reported to have good knowledge in both control and intervention groups. A community-based study in India among men showed that 70% had good knowledge of exclusive breastfeeding and weaning time [34]. However, the finding in this study is at variance with that of Adenike et al. [1] where the knowledge of men regarding maternal health care was poor. Another study in Kaduna State also reported poor knowledge of maternal health care among men (52.6%) [5]. The disparity seen between the findings of this study and that of Adenike et al. [1] and Butawa et al. [5] may be due to differences in the components of PRC assessed, as this study went a step higher to include abortion and post-abortion care. This difference may also be associated with disparity in the content and structure of the questionnaire used. It can also be as a result of differences in the cut-off point used to categorise respondents into good and poor knowledge. Men’s knowledge in the reproductive health issues of their partners is crucial to taking the right decisions and at the same time strengthen their support and care for them.

Almost all the respondents (95.0%) from this study were aware and knew about ANC. This corroborates findings from previous studies [1, 16, 28]. Antenatal care coverage has improved in Nigeria and Africa at large [17]. However, to achieve the full life-saving potential that ANC promises for women and babies, a minimum of eight visits are required to provide adequate contact with healthcare providers [40].

In this study, one of the questions asked to assess knowledge of pregnancy related issues during postnatal period was the timing of recommencing sex after delivery. Only few men (36.8%) knew when sex should recommence. According to Glazener [9], the median time for restarting sexual intercourse was reported to be 6 weeks after delivery. Following the birth of a child many changes occur ranging from physical to emotional and hormonal. These changes include painful sexual intercourse due to healing stitches, low mood and depression; and physiological changes due to breastfeeding or contraception [10]. All of these can affect the woman’s sexual needs and impact on her relationship with her partner. In Nigeria as in many other African countries, men are the initiator of sexual intercourse. It is therefore important for men to know and be well informed about when sexual intercourse should recommence after delivery. Knowledge of newborn care among respondents was good; this did not agree with the finding of Nasreen et al. [28] where knowledge of men regarding wrapping the newborn (13.8%), cutting (29.0%) and tying the cord (19.4%) in a sterile manner were overall poor. The disparity might likely be due to the fact that this study did not ask specific questions related to essential newborn care as largely documented by Nasreen et al.

The report of men’s perception regarding their roles in pregnancy related care in this study showed that almost all the respondents thought they had a role to play during pregnancy, postnatal and newborn care. This is contrary with the finding of Kadam and Payghan [13] in a study in India where men perceived involvement in maternal health care as an unnecessary interference. The difference seen may be due to differences in cultural norms and beliefs.

Specifically, almost all the respondents (98.7%) believed that pregnancy is not the woman’s concern alone that men too have important roles to play. However, almost half of the respondents believed that their role was to provide domestic support while close to one-third (26.7%) of the respondents felt financial support was the only role they had to play. These findings are similar to another study on perception, attitude and involvement of men in maternal health care in the south western Nigeria where less than half of the respondents believed their role was providing emotional and moral support; while 2 out of 10 reported financial support as the only role they have to play [1]. However, other studies have reported the expectations of men to include assistance of their partners during obstetric emergencies and to attend clinic visits [6, 23, 24]. One-fourth of the respondents agreed that men should accompany their wives for antenatal care. These findings are in keeping with reports from other studies [1, 4, 24].

Nearly all the respondents believed they had a role to play in the care of their partners during labour and after delivery (postnatal period) and this support finding from previous studies [1, 8]. Regarding the care of the newborn, majority of the respondents (97.5%) from this study agreed that men have roles to play in the care of the newborn even though it is viewed by many as the duty of the woman alone. It is believed that men are supposed to engage in works that the societies considered more serious like provision of basic necessities for the family than the care of children particularly, the newborn.

Overall, more than half (56.9%) of the respondents had good involvement in pregnancy related care. Pregnancy, childbirth and children upbringing are widely considered to be solely the role of women [30] but male involvement in these states is of crucial importance especially in patriarchal societies like Nigeria, given the elevated position of men in these societies [35]. However, most studies have shown that men can play an important role in reducing maternal and infant morbidity and mortality; and thus, improving the overall wellbeing of the mother and child [13]. The support of the husband has been reported as a good predictor of future practice and continued use of family planning [32]. As far back as 1984, a study done in Philippines showed that the continuation rate of family planning among women whose husbands supported their contraceptive practice is much higher than those whose husbands did not support [32]. Also, Kalembo et al. [14] showed that male partner’s involvement has been seen to increase the uptake of PMTCT services.

Although men are not direct beneficiaries of pregnancy related care services, their understanding, participation and support are crucial in order for women to access basic pregnancy related care services. Although many other factors contribute to increased maternal and infant morbidity and mortality in the developing countries, low male involvement is a major determinant and their participation has been shown to be vital for their reduction [19, 21, 22, 41]. Despite the average involvement of men in pregnancy related care in this study, their involvement in performing care tasks such as accompanying their partner for antenatal and postnatal care; assisting in changing diaper of the baby was poor. The men in this study reported more involvement in providing money and food; and deciding for the partner to attend antenatal care clinic visits. Other researchers in similar studies on men’s role and involvement in maternal health care have documented similar findings [1, 3, 13, 24, 28]. Only 1 out of 5 respondents were involved in following their wives for antenatal care visits in this study and this is in keeping with findings from other studies [1, 4, 13, 24, 32].

The variation in level of support across domains may indicate a difference between men’s ability to be involved in socioculturally acceptable ways (such as directing a female partner to behave in a particular way) compared with more stigmatised ways (such as being absent from paid work to attend maternal and child health services).

Close to two-third (60%) of the respondents accompanied their wives in labour, this is similar to findings in a study carried out in India where majority (87.5%) of the husbands were present during their wives’ delivery [32]. Only 19.9% of the respondents were involved in following their wives to the postnatal clinic. This corroborates findings of Sadhana et al. [32] in India. Almost all of the respondents in this study reminded their wives to attend ANC clinic and PNC clinic visits. This is in keeping with findings from previous studies ([1, 7]; and [28]). A huge gap is observed in the proportions of men who accompanied their partners during intra-natal and postnatal care visits as participation in postnatal care visits was abysmally low. This might be a reflection of their level of understanding in the care mothers receive during these unique visits. This calls for more health promoting activities to improve men’s awareness and participation in postnatal care of their wives and children. At every stage of women’s care in pregnancy related matters, men’s involvement cannot be over-emphasized as there are decisions and activities necessitating the men’s support.

Majority of the respondents were much involved in reminding their partners about activities and making money available for pregnancy related activities, a finding similar to Iliyasu et al. [11] in a study on male participation in maternity care in the northern part of Nigeria. This study found that half (51.0%) of the respondents were involved in newborn care which is at variance with the findings of Kadam and Payghan [13] where very few men were involved in day to day activities of childcare and only 4% helped in feeding the child, 2.67% helped in dressing or bathing the child. The disparity in our study and Kadam et al. may be attributed to the differences in the contents of newborn care assessed. This study considered diaper change and help to ensure mother is comfortable during breastfeeding. Kadam et al. considered day to day activities of childcare like feeding, bathing and dressing for the baby. The poor involvement reported in different domains of pregnancy related care are similar with reports from other studies [1, 4, 7, 23, 24, 28].

Male involvement in pregnancy related care is affected by many factors. Job demand was majorly reported as one of the factors restricting men’s involvement in the care of their partner. Most men are breadwinners in their families and this may account for one of the reasons why they are naturally attached to their jobs and they hardly want to be absent from work. A study in the US to determine what men and women value at work also alluded to this. The study showed that men valued pay, money, and benefits, as well as power, authority, and status more than their female counterparts [31]. In the same study it was shown that women valued friends and relationships, recognition and respect, communication, fairness and equity, teams and collaboration, family and home more than men did.

Some respondents in this study reported social stigma as the factor restricting men from being involved in pregnancy related care. In patriarchal societies like Nigeria, people tend to gossip and look down on men who accompany their spouses to antenatal and postnatal clinic. These findings are in keeping with the findings from a study on male involvement in care and support during pregnancy and childbirth in the Gambia [33]. The Gambian also documented that husbands’ limited time with their spouses in ANC clinic was further complicated by long waiting time for antenatal services.

The findings of this research on bivariate analysis indicated that involvement in pregnancy related care can be explained to a large extent, by factors relating to religion, ethnicity, educational status and knowledge of pregnancy related care. This study revealed that men who were Christians, of Igbo, Hausa and Edo tribes; who had tertiary education; and those with good knowledge of pregnancy related care were more likely to be involved in pregnancy related care. These findings are consistent with findings from previous studies ([3, 11, 25]; and [32]). In the study conducted by Bhatta et al., on involvement of males in antenatal care, birth preparedness, exclusive breastfeeding and immunizations for children in Kathmandu, Nepal revealed men with higher age, uneducated or had primary level of education, had higher income, had formal employment and came from non-indigenous ethnicities were found to be associated with involvement in ANC visit. This shows that religion, cultural norms, level of education and knowledge of PRC, have a great influence on the involvement of male partners in PRC.

### Limitations

This study did not utilize qualitative approach to further explore perception of men regarding their involvement in PRC. Also, men’s willingness to be involved in PRC was not assessed. Being a cross-sectional study, causal relationship could not be established. In addition, the association between knowledge of pregnancy related care and involvement in pregnancy related care could be a complex one. In as much as knowledge of PRC can influence men’s involvement, men who are more involved may likely have more knowledge as well, giving a plausibly a bidirectional relationship between knowledge of PRC and involvement in PRC. This study explored analysis using knowledge of PRC as the explanatory variable and various levels of involvement as the dependent variables. This study was carried out in an urban area in Ibadan hence findings cannot be generalized for the rural area. Predictors of male involvement could not be explored because only few factors were significant on bivariate analysis. Most of the men were of low socio-economic status, hence, there may be a need to explore men in the high socio-economic class.

## Conclusions

The awareness of respondents regarding pregnancy related care was very high but there were gaps in knowledge, perception and involvement in pregnancy related care. Varying levels of involvement were observed in different domains of pregnancy related care such that a high involvement was reported in the areas of reminding and financial support. Whereas, involvement in terms of performing care tasks like accompanying the partner for clinic visits was very low among respondents. Factors like religion, ethnicity, and level of education; and knowledge of pregnancy related care were associated with men’s involvement in pregnancy related care. Job demand, social stigma and long waiting time at the health facilities were reported as inhibiting factors to men’s involvement in pregnancy related care.

## Recommendations

There is need for reproductive health policy review to strongly emphasize the need for involvement of male partners in reproductive health issues including PRC. Public enlightenment programs about PRC should also be organised for men, and they should also be involved in the design and implementation of maternal health services. Health education of men regarding pregnancy related care should include specific issues like accompanying their partners for clinic visits and supporting newborn care. Male involvement should be included in the school curriculum of young children and adolescents. Involvement of religious and community leaders in reproductive health programs to serve as “change advocate” on male partners’ involvement in PRC is also advocated. Health system should be re-structured to create an enabling environment for men to be involved in the care of their pregnant partners by reducing waiting time in the clinics and having education sessions for expectant fathers amongst others. Men will also benefit from paternity leave and specific reproductive health services should be targeted at them to address both their reproductive health concerns and that of their partners.

## Data Availability

The data will be uploaded if needed by the reviewer.

## Abbreviations

ANC: Antenatal Care
ICPD: International Conference on Population and Development
JHPIEGO: Johns Hopkins Program for International Education in Gynecology and Obstetrics
LGAs: Local Government Areas
NDHS: National Demographic Health Survey
PHC: Primary Health Care
PMTCT: Prevention of Mother to Child Transmission
PNC: Post-natal Care
PRC: Pregnancy Related Care
SPSS: Statistical Package for Social Sciences
SRH: Sexual and Reproductive Health
UNFPA: United Nations Population Fund
WHO: World Health Organization
